# Circ-Vps41 positively modulates Syp and its overexpression improves memory ability in aging mice

**DOI:** 10.3389/fnmol.2022.1037912

**Published:** 2022-12-02

**Authors:** Yibo Li, Hongfang Wang, Yanjing Gao, Runjiao Zhang, Qing Liu, Wenmeng Xie, Ziyu Liu, Dandan Geng, Lei Wang

**Affiliations:** ^1^Department of Human Anatomy, Institute of Medicine and Health, Hebei Medical University, Shijiazhuang, Hebei, China; ^2^The Key Laboratory of Neural and Vascular Biology, Ministry of Education, Hebei Medical University, Shijiazhuang, Hebei, China

**Keywords:** aging, circ-Vps41, miR-24a-3p, synaptophysin, learning and memory

## Abstract

**Introduction:**

Age is an established risk factor for neurodegenerative disorders. Aging-related cognitive decline is a common cause of memory impairment in aging individuals, in which hippocampal synaptic plasticity and hippocampus-dependent memory formation are damaged. Circular RNAs (circRNAs) have been reported in many cognitive disorders, but their role in aging-related memory impairment is unclear.

**Methods:** In this study, we aimed to investigate the effects of circ-Vps41 on aging-related hippocampus-dependent memory impairment and explore the potential mechanisms. Here, D-galactose was used to produce a conventional aging model resulting in memory dysfunction.

**Results:**

Circ-Vps41 was significantly downregulated in D-galactose-induced aging *in vitro* and *in vivo*. The overexpression of circ-Vps41 could upregulate synaptophysin (Syp), thereby promoting the synaptic plasticity and alleviating cognitive impairment in aging mice. Mechanistically, we found that circ-Vps41 upregulated Syp expression by physically binding to miR-24-3p. Moreover, the miR-24-3p mimics reversed the circ-Vps41 overexpression-induced increase in Syp expression.

**Discussion:**

Overexpression of circ-Vps41 alleviated the synaptic plasticity and memory dysfunction via the miR-24-3p/Syp axis. These findings revealed circ-Vps41 regulatory network and provided new insights into its potential mechanisms for improving aging-related learning and memory impairment.

## Introduction

Aging impacts a variety of brain structure and functions, and makes elderly individuals more likely to develop neurodegenerative diseases and cognitive impairments (Yousef et al., 2019). Loss of memory during normal aging has a serious impact on the quality of life of the elderly, which is a heavy burden to both patient’s family and the whole society (Xiang et al., 2018). Although much is known about the development of cognitive processes in the brain (Johnson and Munakata, 2005; Gomes da Silva and Arida, 2015; Oschwald et al., 2019), the study of the molecular mechanisms governing aging-related memory decline is still less explored.

Synaptic plasticity is considered to be an important basis for the formation of learning memory (Koh et al., 2010; Anacker and Hen, 2017). Synapses are the important functional units of learning and memory in the brain (Guo et al., 2019). Loss of dendritic spines directly correlates with loss of synaptic function and memory deficits (Cissé et al., 2017; Wang et al., 2017). Synaptophysin (Syp) is an abundant synaptic vesicle membrane protein involved in synaptic vesicle exocytosis and has been commonly used to represent synaptic plasticity in the presynaptic region (Osimo et al., 2019). Studies have shown that Syp expression decreases significantly in the aging brain (Eastwood et al., 2006; Canas et al., 2009; Marschallinger et al., 2015). Exercise ameliorated decreased expression of Syp in hippocampus and could significantly improve the learning and memory capacity in aging process (Chen et al., 2020). The same phenomenon was observed in the hippocampus of aging mice induced by D-galactose (Li et al., 2019; Garcez et al., 2021). Despite Syp plays an important role in memory impediment and progression to aging, the mechanisms that regulate its expression, and contribute to its increased expression in hippocampus, are not well understood.

In recent years, circular RNAs (circRNA) have been identified as important molecules in gene-expression regulation at the post-transcriptional level (Zhou et al., 2020). The hippocampus has been shown to express specific regions of circRNAs in the mouse brain (Rybak-Wolf et al., 2015). Some studies have shown that circRNAs are specifically located in dendrite (You et al., 2015), axons (Qian et al., 2022) and synapses (Curry-Hyde et al., 2020), regulating synaptic plasticity (Xu et al., 2020). CircRNAs also play a causative role in aging and longevity, whose levels are also altered in an age-dependent manner across specific human organs and tissues (Kim et al., 2021). Circ-Vps41 is derived from the circularization of exons 8, 9 and 10 of the Vps41 gene and is preferentially localized in the cytoplasm (Zhang et al., 2022). In our previous study, we examined the circRNAs expression profile in the hippocampus of Nrf2 knock-out mice and found circ-Vps41 acted as a miR-26a-5p sponge to regulate CaMKIV, which could improve the learning and memory impairment of Nrf2 knock-out mice (Zhang et al., 2019, 2022). While a few studies have been reported on the roles of circRNAs in aging, their functions in aging-related memory impairment and the underlying mechanism have not been well elucidated.

In this study, we aimed to investigate the role of circ-Vps41 in aging-related memory and explored its potential mechanisms. We used D-galactose to produce a conventional aging model (Azman and Zakaria, 2019) and found that the expression of circ-Vps41 was decreased in D-galactose-induced aging cell and animal model. To further reveal the role of circ-Vps41 in aging-related memory and learning, we demonstrated that circ-Vps41 can promote the expression of Syp by sponging miR-24-3p. Finally, the ability of memory were enhanced in D-galactose-induced aging mice, thus potentially representing a new therapeutic target for improving memory during aging.

## Materials and methods

### Cell culture

Immortalized mouse hippocampal HT22 cells were maintained in Dulbecco’s modified Eagle’s medium (DMEM; cat#: PM150210, Procell, China) with 10% fetal bovine serum (FBS; cat#: FB25015, Clark Bioscience, United States) and a 1% penicillin/streptomycin solution in an H_2_Osaturated 5% CO_2_ atmosphere at 37°C. The cells were digested with 0.25% trypsin at 37°C. The cells were cultured for another day in 6-well plates for protein/mRNA assays.

### CCK-8

Cell Counting Kit-8 (cat#: C0037, Beyotime, China) was used to evaluate cell viability. HT22 cells were seeded in a panel of five 96-well plates at a density of 8,000 cells per well. After the cells adhered to the wall, the culture medium was withdrawn, and DMEM complete medium containing a different concentration of D-galactose (50, 75, and 100 mM) was added for 5 days. From five duplicate 96-well plates, the relative cell viability was measured by an assay kit at the same time every day according to the manufacturer’s instructions. The absorbance at 450 nm was measured with a microplate reader. The experiment was repeated three times. The linear correlation between the average absorbance and the relative content was analyzed. The precision of the method was evaluated with the mean relative SD of three measurements. Repeated measures and multivariate analysis of variance (ANOVA) of the general linear model in SPSS were used to assess the significant differences among different groups.

### β-galactosidase staining

A senescent cell staining kit (cat#: G1580 Solarbio, Beijing, China) was used to perform the senescence-associated-β-galactosidase (SA-β-gal) assay according to the manufacturer’s protocol. After β-galactosidase staining of HT22 cells or frozen sections of mouse hippocampus, images were acquired with a light microscope (Leica, DM2000 LED, Germany). Total cells were counted in three random fields per culture dish to determine the percentage of SA-β-gal-positive cells. The images were analyzed using Image-Pro Plus 6.0 to determine the optical density for quantitative analysis.

### Establishment of an HT22 senescent cell model

By mixing the 10.8 g D-galactose (cat#:G0008 TCI, Tokyo, Japan) powder with 10 ml of PBS, a 1 M suspension was taken appropriately and diluted to 50, 75, and 100 mM using DMEM high glucose complete medium. The cells were plated onto 96-well plates for the CCK-8 assay and 24-well plates for β-galactosidase. Ultimately, the duration of 75 mM for 2 days was considered the optimal protocol for successfully modeling HT22 senescent cells. The cells were tested by qRT-PCR or western blot.

### RT-qPCR

To detect the expression levels of circ-Vps41 and Syp mRNA, total RNA from cells or dissected hippocampal tissues was extracted using an animal tissue/cell total RNA kit (cat#: ZP404, ZOMANBIO, China) according to the manufacturer’s instructions. First-strand cDNA was generated from 1 μg of total RNA using HiScript III RT SuperMix for qPCR (cat#: R323, Vazyme, China) with the following three-step incubation: 42°C for 2 min, 37°C for 15 min, and 85°C for 5 s. RT-qPCR was performed using ChamQ Universal SYBR qPCR Master Mix (cat#: KT201, Vazyme, China). β-actin was used for normalization. Samples were incubated at 95°C for 15 min, followed by 40 cycles of denaturation at 95°C for 10 s, annealing at 53°C for 20 s, and cDNA extension at 72°C for 20 s. After the amplification cycles, a final extension step at 72°C was performed for 10 min. All experiments were performed in triplicate. The threshold cycle (Ct value) was recorded by a QuantStudio^™^ 6 Flex Real-Time PCR System (REF#: 4484642, Applied Biosystems, United States). The primer sequences used in this study were circ-Vps41 forward primer: 5′-ACACTCATTATTGGCTGGGGAACTT-3′, reverse primer: 5′-CACACTCCTTATGTTTCCTTCCCCT-3′; Syp forward primer: 5′-CAGTTCCGGGTGGTCAAGG-3′, reverse primer: 5′-ACTCTCCGTCTTGTTGGCAC-3′; β-actin forward primer: 5′-TCATCACTATTGGCAACGAGCGGT-3′, reverse primer: 5′-GTGTTGGCATAGAGGTCTTTACG-3′. Two-tailed t tests (2 groups) or one-way ANOVA (3 groups) were used for the statistical analyses, and the relative expression of RNAs was analyzed with the 2^–ΔΔCt^ method.

To detect the expression level of miR-24-3p, miRNAs were extracted from cells using the miRcute miRNA isolation kit (cat#: DP501, Tiangen, China) according to the manufacturer’s instructions. cDNA was generated using the miRcute Plus miRNA First-strand cDNA Kit (cat#: KR211, TIANGEN, China), which was performed according to the manufacturer’s protocols. RT-qPCR was performed using ChamQ Universal SYBR qPCR Master Mix (cat# KT201, Vazyme, China). The forward primers for miR-24-3p (CD202-0009) were acquired from Tiangen Biotech. U6 was quantified as a control to normalize differences in the total RNA levels. The statistical analyses were the same as described above.

### Western blot

For preparation of protein samples, the cells or dissected hippocampal tissue were homogenized or lysed in RIPA buffer (cat#: R0020, Solarbio, China) supplemented with PMSF (100 mM; cat#: P0100, Solarbio, China). After ultrasound sonication and incubation on ice for an hour, the protein samples were cleared by centrifugation at 12000 × *g* for 20 min at 4°C, and the supernatant was collected. Then, the protein concentration was evaluated using a BCA Protein Assay reagent kit (cat#: PC0020, Solarbio, China) and an Infinite F200 (TECAN, Switzerland) plate reader. Protein samples were mixed with 5 × loading buffer (cat#: P1040, Solarbio, China). After boiling, 30 μg of protein was separated by a 10% SDS polyacrylamide gel and electrotransferred onto polyvinylidene fluoride membranes. The membranes were then blocked with 5% milk in TBST (0.1% Tween-20) and incubated with the following: rabbit anti-postsynaptic density-95 (PSD95; 1:2000, cat#: ab18258, Abcam, United States), rabbit anti-synaptophysin (Syp; 1:2000, cat#: CY5273, Abways, China), and rabbit anti-β-actin (1:600000, cat#: AC026, ABclonal, China). Detection and analysis were performed with the Odyssey system (Odyssey, cat#: 987-07708, LI-COR Biosciences, United States). Two-tailed *t*-tests (2 groups) or one-way ANOVA (3 groups) was used for the statistical analyses.

### Knockdown of circ-Vps41 with RNA interference in HT22 cells

Small interfering RNA (siRNA) targeting circ-Vps41 (si-circ-Vps41) and negative control (NC) provided by GenePharma Inc. were used for transfection in the cells at 60% confluence with the assistance of Lipofectamine 3,000 (cat#: L3000015, Thermo Fisher Scientific, United States). Each siRNA-Lipofectamine 3,000 premix was added to the six-well plates, with a final concentration of siRNA of 40 nM/well and a volume fraction of Lipofectamine 3,000 of 1/500. The cells were cultured for another 2–4 days and tested by qRT–PCR or western blot.

### Overexpression of circ-Vps41 and miR-24-3p in HT22 cells

To overexpress circ-Vps41, circ-Vps41 mouse cDNA ORF Clone, as in our previous study (Zhang et al., 2022), was used with pcDNA3.1 (+) circRNA mini vector as the backbone vector. To overexpress miR-24-3p, miR-24-3p mimics provided by GenePharma Inc. were employed. With the assistance of Lipofectamine 3,000 (cat#: L3000015, Thermo Fisher Scientific, USA), the recombinant expression plasmids, miR-24-3p mimics or corresponding control vectors were transfected into HT22 cells separately, which were then cultured for another 2–4 days and tested by qRT-PCR and western blot.

### Animal model

To establish the animal model of D-galactose-induced aging, 4-month-old male ICR mice were treated with 150 mg of D-galactose (cat#:G0008 TCI, Tokyo, Japan) per kg of body weight daily for 6 weeks (D-gal group). Meanwhile, mice in the control group were treated with the same dose of physiological saline. All animal experiments complied with the regulations of the Animal Welfare Act of the National Institutes of Health Guide for the Care and Use of Laboratory Animals (NIH Publication No. 85–23, revised 1996) and were approved by the ethics committee of Hebei Medical University (IACUC-Hebmu-Glp-2,016,017). All investigators were blinded. The study was not preregistered.

### Object recognition test (ORT)

Object recognition test was used to assess learning and memory abilities based on the tendency of mice to discriminate a familiar from a new object (Hong et al., 2020). The test was completed in 3 days and consisted of three phases: habituation, training, and testing. Initially, all experimental mice were allowed to adapt in the test room for 24 h and habituated to an open-field box for 20 min. During the training phases, each mouse was allowed to freely explore two identical objects for 5 min. After 2 and 24 h, mice were returned to the area with one familiar object and one novel object for testing. The recognition index for the new object was evaluated (RI = TN/(TF + TN) × 100%). TN: the time to explore a new object, TF: the time to explore a familiar object. One-way ANOVA was used for the statistical analyses.

### Morris water maze task (MWM)

The MWM test was conducted to evaluate the aging mice of spatial learning and memory as described in a previous study (Li et al., 2016). The test was completed in 7 days using a cylindrical tank and a video capture system. The maze was divided into four quadrants with a platform placed in one quadrant. On the first day, each mouse was adapted into the test room and swam for 2 min to find a round platform above water level. After excluding a few mice that could not adapt to the swimming tank environment, 34 mice were used for this test. During 5 consecutive days of continuous training, the mice were trained to locate a hidden platform in the water maze, four trials per day (with a 30 min interval) from 14:00 to 20:00 pm. If the mouse failed to find the platform within 60 s, it was allowed to stay on the platform for 20 s. The swimming path, the time used to find the platform (average latency), the percentage of time spent in the target quadrant, and the average velocity were recorded by a video capture system of Smart software. On the 7th day, the platform was removed from the tank. The mouse was allowed to swim freely for 60 s and the numbers of the platform crossing was recorded by video capture system. Repeated measures and multivariate analysis of variance (ANOVA) process of the general linear model in SPSS was used to assess the significant differences among different groups.

### Virus stereotaxic injections

A DNA sequence that amplifies circ-Vps41 expression was incorporated into the adeno-associated virus (AAV) vector (pHBAAV-CMVcircRNA-EF1-ZsGreen), which contains a green fluorescent protein (GFP) sequence and the recombinant vector was named AAV-circ-Vps41. The same AAV vector containing only GFP was used as a control and named AAV-GFP. Both vectors were produced by Hanbio Biotechnology (Shanghai, China).

For stereotactic brain injection, we defined stereotactic injection coordinates, in the lateral skull stereotaxically at posterior 1.89 mm, lateral 2.29 mm, and ventral 1.65 mm relative to bregma, for the CA1 region of hippocampus according to the mouse brain atlas in software. With 1% pentobarbital sodium (0.1 ml/20 g)-anesthetized D-gal group mice (*n* = 20) and control group mice (*n* = 10) were placed in a stereotaxic apparatus. AAV-circ-Vps41 or AAV-GFP (1 μl) was injected at a rate of 0.1 μl/min using a microinjection system (cat#: 78–1,311 Q, KD Scientific, United States). The needle was kept in place for 15 min before withdrawal, as in our previous study (Zhang et al., 2022).

### Golgi-Cox staining

For morphological quantification of dendritic spine density in hippocampal neurons, Golgi-Cox staining (cat#: GMS80020.1, GenMed, United States) was utilized. After being fixed with Golgi immersion solution for 14 days, hippocampal tissue sections 100 μm thick were cut at room temperature using a vibrator (Leica VT100S, Germany), dehydrated with a gradient of 50, 75, 95, to 100% ethanol, cleared in xylene, and sealed with neutral balsam. Lastly, the slides were viewed in detail with a light microscope (Leica DM2000 LED, Germany). Five segments of 10 μm (or longer) apical dendrites were randomly selected from each pyramidal neuron for inspection to quantify the density of spines. Secondary and tertiary apical dendrites were selected for quantitative analysis in the CA1 region of the hippocampus. The number of dendritic spines was independently quantified using FIJI ImageJ software^1^ by two different investigators, and the results were cross-checked to preclude systematic analytical errors. One-way ANOVA was used for the statistical analyses.

### Immunohistochemical staining

Frozen sections of mouse hippocampus (10 μm) were rehydrated in a descending concentration of ethanol following antigen retrieval in citrate buffer. The sections were treated according to the manufacturer instructions using SP-9001 detection kit (cat#: SP-9001, ZSGBBIO, China). After the endogenous peroxidase activity was quenched with 0.3% H_2_O_2_, the sections were blocked in FBS at 37°C for 30 min. Then, the sections were incubated in rabbit anti-Syp antibodies (1:300, cat#: CY5273, Abways, China) and rabbit anti-PSD95 (1:200, cat#: ab18258, Abcam, United States) overnight at 4°C. Immunodetection was carried out with the diaminobenzidine–horseradish peroxidase (HRP) reaction system. Images were analyzed and processed using the software Image-Pro Plus 6.0.

### Dual-luciferase reporter assay

Luciferase reporter assay was applied to verify whether miR-24-3p can target circ-Vps41 or Syp mRNA 3′UTR directly. Firstly, based on the putative miR-24-3p binding sites in the circ-Vps41 (circ-Vps41-WT) and Syp mRNA 3′UTR (Syp-WT), the circ-Vps41 and Syp mRNA 3′UTR with mutated miR-24-3p binding sites (circ-Vps41-Mut, Syp-Mut) were generated. Luciferase reporter of psi-CHECK2 vectors containing mutated sites was constructed by General biosystems (Anhui, China). Subsequently, the recombinant luciferase reporter plasmids, miR-24-3p mimics, or circ-Vps41 overexpression vector was transferred into 293A cells for 24 h. Finally, the luciferase activities were measured as described above. The relative fluorescence activity was calculated as Renilla fluorescence/Firefly fluorescence. One-way ANOVA was used to assess the interactions between circ-Vps41/Syp mRNA 3′ UTR and miR-24-3p.

### Fluorescence *in situ* hybridization (FISH)

RNA FISH was performed using RNA FISH Kit (cat#: D-2922B, EXONBIO, Guangzhou, China; cat#: C10910, RiBo, Guangzhou, China) in frozen sections of mouse hippocampus. The probe for miR-24-3p was produced by Exonbio Lab (Guangzhou, China) and circ-Vps41 was produced by RiBo Lab (Guangzhou, China; Zhang et al., 2022). FISH was used to detect the expressions of miR-24-3p and circ-Vps41. Hybridization with probes were conducted at 37°C. Following the staining with DAPI, the confocal microscope (Olympus FV1000, Tokyo, Japan) was used to capture the images.

## Results

### circ-Vps41 expression was downregulated in the D-galactose-induced aging model

**Figure 1:**
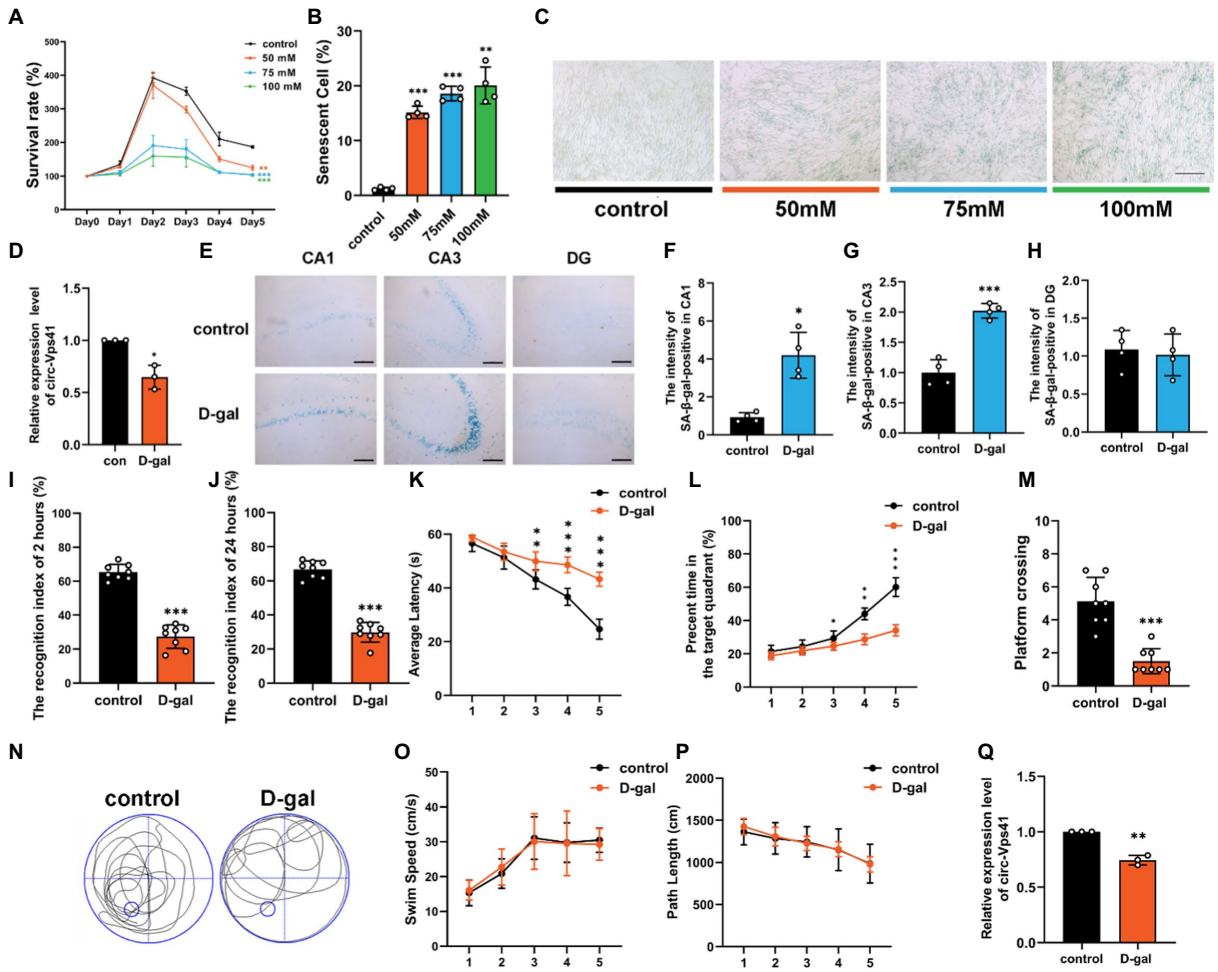
circ-Vps41 expression was downregulated in the D-galactose-induced aging model. **(A)** Cell growth assays (CCK8) demonstrate that treatment with D-galactose leads to decreased viability in HT22 cells, which **(B,C)** is confirmed by the high percentage of senescent cells in β-galactosidase staining (scale bars = 200 μm). ANOVA was used to test for differences among all groups. **(D)** The expression level of circ-Vps41 was tested by qRT–PCR in HT22 cells in the control (con) and 75 mM D-galactose (D-gal) groups. The con group was normalized to 1. **(E)** SA-β-gal-positive cells could be observed in the hippocampus at CA1, CA3 and DG areas of D-galactose-induced aging mice. (Scale bars = 100 μm). **(F–H)** Digitized for analysis by ImageJ software, stained levels of SA-β-gal-positive cells for D-gal group at CA1, CA3 and DG areas of the hippocampus in the ipsilateral side compared to the control groups (*n* = 4 for control group, *n* = 4 for the D-gal group). **(I,J)** ORT test of mice at 2 and 24 h. **(K)** Time taken by mice to reach the platform (average latency) during the training trials. **(L)** Percentage of time in the target quadrants of mice during the training trials. **(M,N)** Numbers of platform crossings of mice and representative swimming paths in the 7-day MWM. **(O,P)** Average swim speed and total path length by mice to reach the platform during the training trials (*n* = 8 for the control group, *n* = 8 for the D-gal group). **(Q)** The expression level of circ-Vps41 was tested by qRT–PCR in the hippocampus of mice in the control and D-gal groups (*n* = 3 for the control group, *n* = 3 for the D-gal group). The control group was normalized to 1. Data are shown as the mean ± SEM. *p* values, two-tailed *t*-test, one-way ANOVA. ^*^p < 0.05, ^**^p < 0.01, ^***^p < 0.001.

Our previous work analyzed the circRNA profiles in the hippocampus of wild-type and Nrf2 knock-out mice using a whole transcriptome microarray (Zhang et al., 2019), and the lower expression of circ-Vps41 was observed in Nrf2 knock-out mice, which showed decreased synaptic plasticity (Zhang et al., 2022). To further investigated the effects of circ-Vps41 on aging-related hippocampus-dependent cognitive impairment. In the present study, an *in vitro* aging model was successfully established by treating HT22 cells with 75 mM D-galactose for 2 days. The cells showed gradual reduction of cell viability by CCK-8 assay (Figure 1A), and the percent of senescent cells significantly increased by β-Galactosidase staining (Figures 1B,C). Consistent with this finding, we observed that the expression of circ-Vps41 was significantly downregulated (Figure 1D).

To validate the observation described above, we established an aging animal model by administering D-galactose for 6 consecutive weeks. β-Galactosidase staining experiments demonstrated that senescent cells substantially accumulated in the CA1 and CA3 regions of the hippocampus in D-galactose-induced aging mice compared with control group (Figures 1E–H). To explore cognitive function, we evaluated objective recognition memory with the ORT. After 2 and 24 h of the training period, the test session was performed, in which a familiar object was replaced by a novel object. D-galactose-induced aging mice had a significantly decreased recognition index compared to control mice (Figures 1I,J). Then, we conducted the MWM test, which is widely used to study hippocampus-dependent spatial learning and memory. The MWM results showed that D-galactose-induced aging mice increased the escape latency (Figure 1K), shortened the percentage of time in the target quadrant (Figure 1L), and performed significantly fewer crossing number than control group (Figures 1M,N). However, there was no difference in average swimming speed and total path between groups (Figures 1O,P).

We next evaluated the expression level of circ-Vps41 in the hippocampus of D-galactose-induced aging mice. qRT-PCR assays showed that circ-Vps41 expression was indeed significantly reduced in D-galactose-induced aging mice compared to control group (Figure 1Q). Taken together, our results confirmed that circ-Vps41 expression was significantly reduced in D-galactose-induced aging model *in vitro* and *in vivo*. In order to explore whether low expression of circ-Vps41 was specific to the hippocampal region or a generalized phenomenon in the central nervous system in D-galactose-induced aging mice. Here, we analyzed the expression of circVps41 in other brain structures (cortex and cerebellum), the results showed that circ-Vps41 expression was also reduced in the cortex, but not in the cerebellum (Supplementary Figure 1A). In addition, we also verified that the circ-Vps41 expression was indeed significantly reduced in 20-month-old natural aging mice, this gave a better availability of D-galactose-induced aging mice (Supplementary Figure 1B).

### Overexpression of circ-Vps41 improves dendritic spine density and Syp expression in D-galactose-induced aging mice

**Figure 2:**
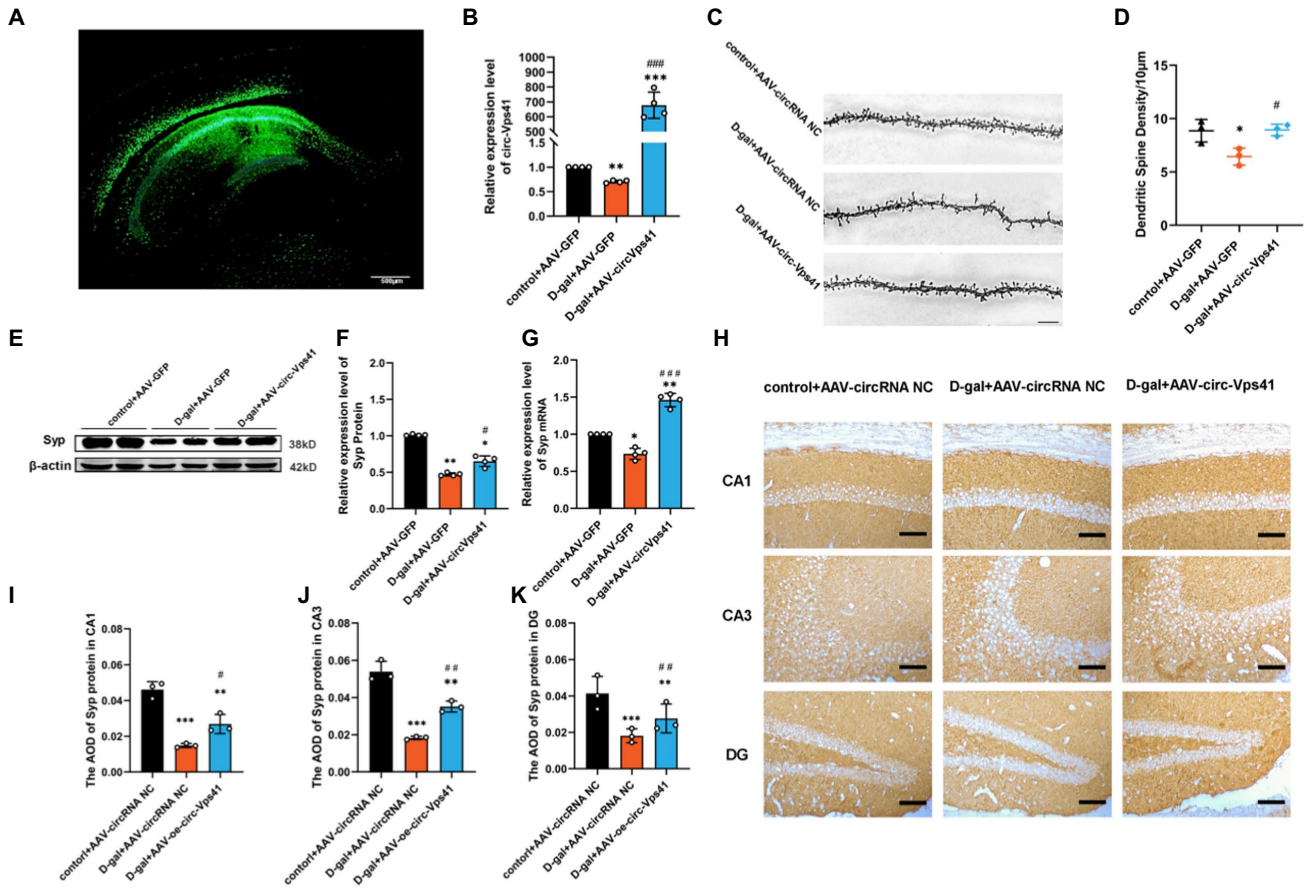
Overexpression of circ-Vps41 improves dendritic spine density and Syp expression in D-galactose-induced aging mice **(A)** Representative image of stereotactic injections in the hippocampal CA1 area. (Scale bars = 500 μm). **(B)** The expression level of circ-Vps41 was tested by qRT–PCR in the hippocampus of mice (*n* = 4 for the control+AAV-GFP group, *n* = 4 for the D-gal+AAV-GFP group, *n* = 4 for the D-gal+AAV-circ-Vps41 group). The control+AAV-GFP group was normalized to 1. **(C,D)** Representative images and quantification of Golgi-Cox staining in the hippocampus of the three groups of mice. (*n* = 3 for the control+AAV-GFP group, *n* = 3 for the D-gal + AAV-GFP group, *n* = 3 for the D-gal+AAV-circ-Vps41 group, scale bars = 10 μm). **(E,F)** Representative images and quantification of Syp in the hippocampus of the three groups of mice were tested by western blot. **(G)** The expression level of Syp mRNA was tested by qRT–PCR in the hippocampus of mice in the three groups. The control + AAV-GFP group was normalized to 1. **(H–K)** Representative images and quantification of Syp in the hippocampus of the three groups of mice were tested by immunohistochemical staining (*n* = 3 for the control + AAV-GFP group, *n* = 3 for the D-gal + AAV-GFP group, *n* = 3 for the D-gal + AAV-circ-Vps41 group, scale bars = 100 μm). ^*^p < 0.05, ^**^p < 0.01, ^***^p < 0.001 vs. control + AAV-circRNA NC group; ^#^*p* < 0.05, ^##^*p* < 0.01, ^###^*p* < 0.001 vs. D-gal+AAV-GFP group; Data shown as the mean ± SEM. *p* values, one-way ANOVA.

To explore the role of circ-Vps41 in the D-galactose-induced aging mice, circ-Vps41-overexpressing recombinant adeno-associated virus (AAV-circ-Vps41-GFP) was synthesized. D-galactose-induced aging mice and control mice received a stereotactic injection of AAV-circ-Vps41-GFP into both sides of the hippocampus (Figure 2A), and its transfection efficiency was detected by qRT-PCR (Figure 2B).

Dendritic spines are specialized structures whose plasticity underlie learning and memory processes (Rochefort and Konnerth, 2012). Thus, it is plausible that the impairments in learning and memory observed D-galactose-induced aging mice are associated with structural and morphological alterations of dendritic spines. In order to verify whether the overexpresion of circ-Vps41 is related to synaptic plasticity, we performed Golgi staining and quantified dendritic spine density in CA1 region of the hippocampus. The density of dendritic spines decreased in D-gal+AAV-GFP group compared with control+AAV-GFP group, and D-gal+AAV-circ-Vps41 group significantly increased the dendritic spines to the control level (Figures 2C,D), but control + AAV-circ-Vps41 group did not increase dendritic spines compared with control + AAV-GFP group (Supplementary Figures 2A,B).

Syp, an integral membrane protein of synaptic vesicles, reflects changes in synapse vesicles, and Syp expression is reduced by synaptic dysfunction (Osimo et al., 2019). There is a close relationship between the changes of Syp in hippocampus and the defects of learning and memory during aging (Kurihara et al., 2008; Afshordel et al., 2014). In the present study, D-galactose treatment significantly decreased the expression level of Syp compared with the control group, and the overexpression of circ-Vps41 increased the deficit of Syp protein and Syp mRNA in aging mice (Figures 2E–G), but the overexpression of circ-Vps41 in control mice did not increase the protein expression of Syp (Supplementary Figures 2C,D). In addition, immunohistochemical staining showed that the Syp expression was significantly reduced by the D-galactose treatment, but this inhibitory effect was reversed after the AAV-circ-Vps41 transfection (Figures 2H–K). These results indicated that circ-Vps41 facilitated dendritic plasticity and Syp expression.

### circ-Vps41 regulates the expression of Syp *in vitro*

**Figure 3:**
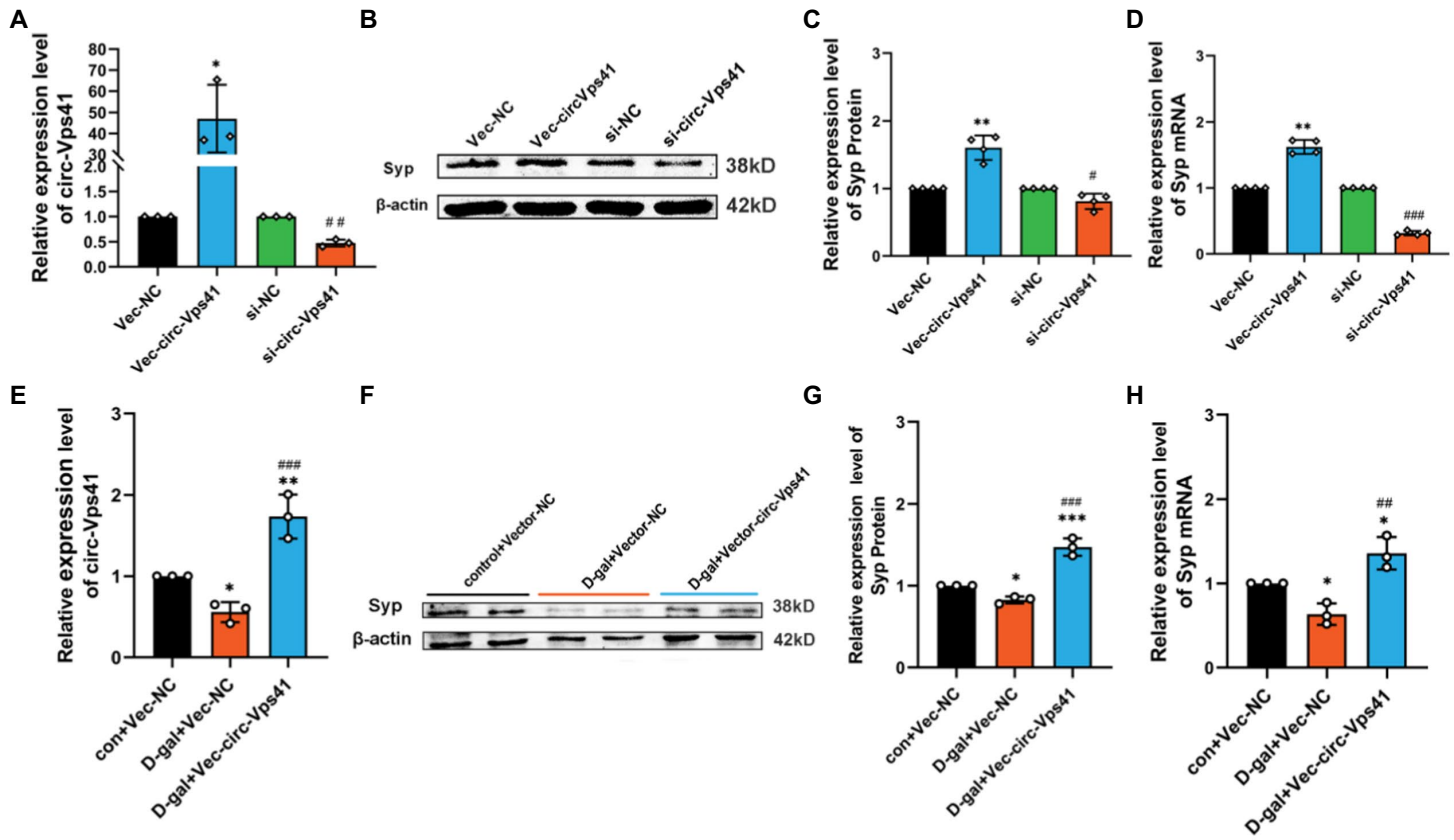
circ-Vps41 regulates the expression of Syp *in vitro* **(A)** The knockdown and overexpression efficiency of circ-Vps41 were measured by qRT–PCR in HT22 cells. **(B,C)** Representative images and quantification of Syp in HT22 cells after knockdown and overexpression of circ-Vps41 were tested by western blot. **(D)** The expression level of Syp mRNA was tested by qRT-PCR in HT22 cells after knockdown and overexpression of circ-Vps41. ^*^p < 0.05, ^**^p < 0.01, ^***^p < 0.001 vs. Vec-NC group; ^#^*p* < 0.05, ^##^*p* < 0.01, ^###^*p* < 0.001 vs. si-NC group. **(E)** The expression level of circ-Vps41 was tested by qRT–PCR after the overexpression of circ-Vps41 in D-galactose-induced aging HT22 cells. **(F,G)** Representative images and quantification of Syp after the overexpression of circ-Vps41 in D-galactose-induced aging HT22 cells were tested by western blot. **(H)** The expression level of Syp mRNA was tested by qRT-PCR after the overexpression of circ-Vps41 in D-galactose-induced aging HT22 cells. ^*^p < 0.05, ^**^p < 0.01, ^***^p < 0.001 vs. con + Vec-NC group; ^#^*p* < 0.05, ^##^*p* < 0.01, ^###^*p* < 0.001 vs. D-gal + Vec-NC group. Data are shown as the mean ± SEM. *p* values, two-tailed *t*-test, one-way ANOVA. The controls group were normalized to 1.

To further confirm the function that circ-Vps41 regulates the Syp expression, we transfected circ-Vps41 overexpressed plasmids, or si-circ-Vps41 into HT22 cells (Figure 3A). These experiments showed that upregulation of circ-Vps41 significantly enhanced the Syp expression of HT22 cells, and knockdown circ-Vps41 inhibited the Syp expression of HT22 cells (Figures 3B–D).

Furthermore, we analyzed the protein expression levels of Syp by Western blot in D-galactose-induced aging HT22 cells. We upregulated circ-Vps41 by transfecting the circ-Vps41 overexpressed plasmids into D-galactose-induced aging HT22 cells (Figure 3E). Syp protein expression were significantly reduced in the 75 mM D-gal group compared with the control group, and circ-Vps41 overexpression reversed the decreasing of Syp protein by the D-galactose treatment (Figures 3F–H).

### circ-Vps41 acts as a sponge of miR-24-3p

**Figure 4:**
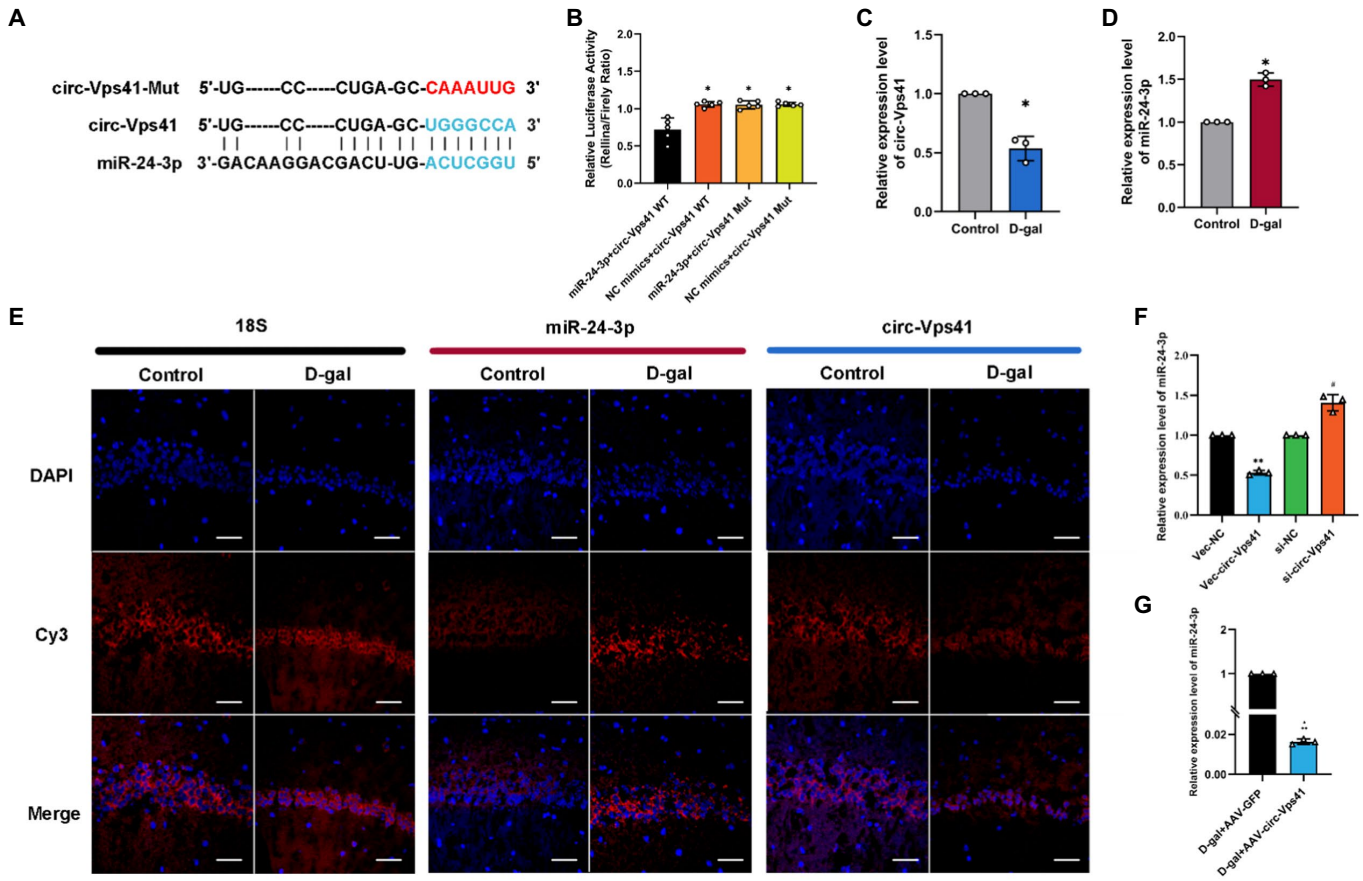
circ-Vps41 acts as a sponge of miR-24-3p. **(A)** Predicted binding sites of miR-24-3p in circ-Vps41; the mutation sequence is shown in red. **(B)** The regulatory effect of circ-Vps41 on the expression of miR-24-3p was verified by dual-luciferase reporter gene detection. **(C–E)** Representative FISH images of circ-Vps41, miR-24-3p, and 18S in the CA1 region of the hippocampus; Scale bars = 50 μm. The Control group was normalized to 1. **(F)** The expression level of miR-24-3p was tested by qRT–PCR in HT22 cells after knockdown and overexpression of circ-Vps41. ^*^p < 0.05, ^**^p < 0.01 vs. Vec-NC group; ^#^*p* < 0.05 vs. si-NC group. The Vec-NC group and si-NC were normalized to 1. **(G)** The expression level of miR-24-3p was tested by qRT–PCR in the hippocampus of D-galactose-treated aging mice after overexpression of circ-Vps41. (*n* = 3 for the D-gal + AAV-GFP group, *n* = 3 for the D-gal + AAV-circ-Vps41 group). The D-gal + AAV-GFP group was normalized to 1. Data are shown as the mean ± SEM. *p* values two-tailed *t*-test, one-way ANOVA. ^*^p < 0.05, ^**^p < 0.01, ^***^p < 0.001.

circ-Vps41 is localized in the cytoplasm based on our previous FISH results (Zhang et al., 2022). It has been reported that circRNAs, which mainly located in the cytoplasm, always function as a sponge of miRNAs (Chen, 2016). Thus, we predicted the potential target miRNAs of circ-Vps41 using RNAhybrid. MiR-24-3p were predicted as a potential target. The putative binding sites of circ-Vps41 are shown in Figure 4A. To elucidate the interaction between circ-Vps41 and miR-24-3p, we inserted the luciferase reporter with wild (circ-Vps41 WT) or mutant-type (circ-Vps41 Mut) sequences that were predicted to be potential binding sites for circ-Vps41 and miR-24-3p. The relative luciferase activity was decreased by miR-24-3p mimics in cells transfected with wild-type constructs, while no significant difference was observed in the mutant group (Figure 4B), indicating that miR-24-3p could directly bind to circ-Vps41.

Subsequently, we analyzed the expression level of circ-Vps41 and miR-24-3p in D-galactose-induced aging model. FISH results showed that circ-Vps41 was significantly reduced and miR-24-3p was significantly increased in D-gal group compared to control group (Figures 4C–E). Further, the qRT–PCR results showed that circ-Vps41 overexpression significantly decreased miR-24-3p expression in HT22 cells. Additionally, we knockdown circ-Vps41 by transfecting the si-circ-Vps41, the expression level of miR-24-3p was significantly increased (Figure 4F). The results revealed that circ-Vps41 overexpression significantly reduced the expression of miR-24-3p in D-galactose-induced aging model (Figure 4G). Taken together, the above results implied that circ-Vps41 serves as a competing endogenous RNA *via* sponging miR-24-3p.

### circ-Vps41 improves Syp expression depending on miR-24-3p

**Figure 5:**
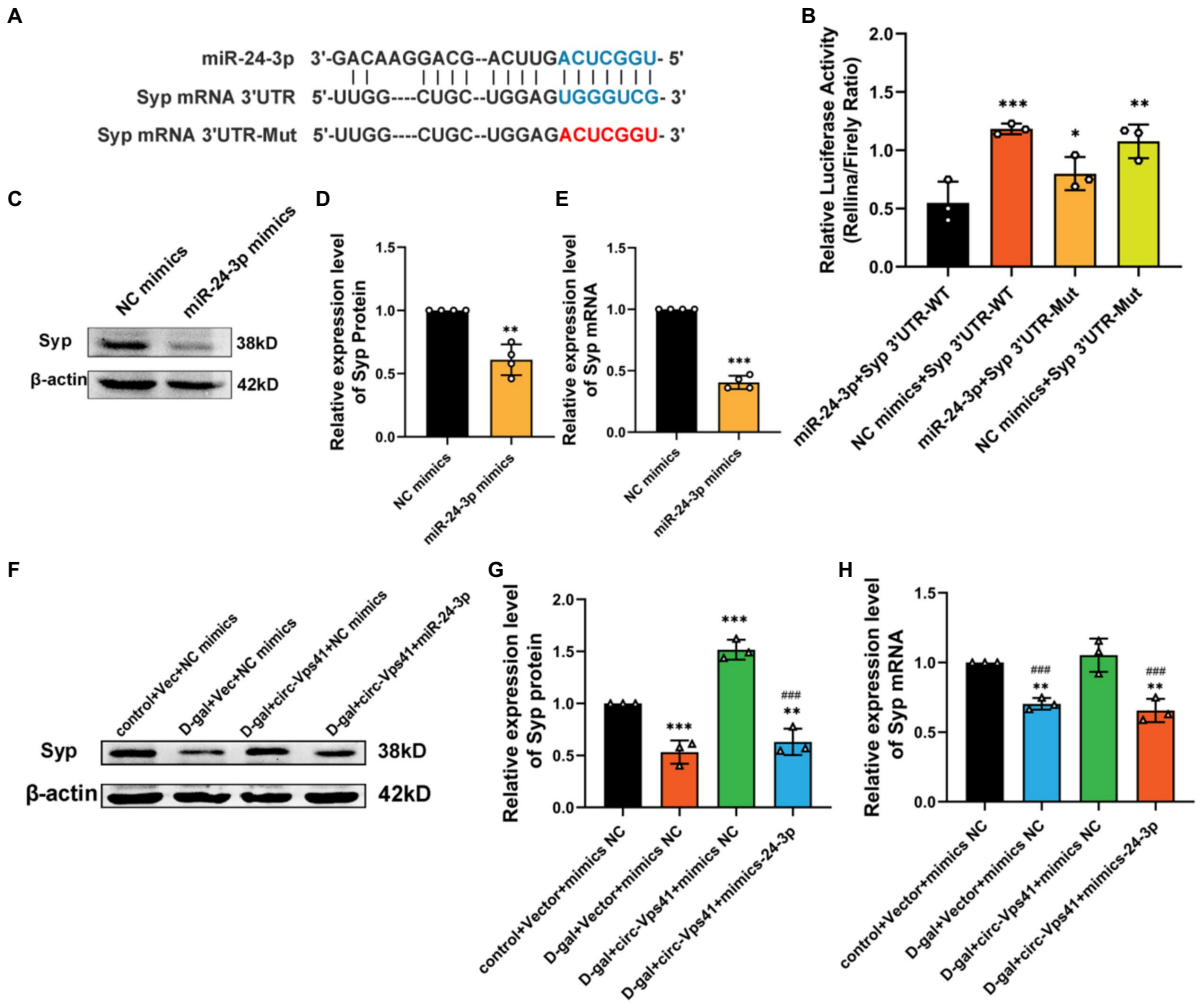
Circ-Vps41 improves Syp expression depending on miR-24-3p. **(A)** Predicted binding sites of miR-24-3p in the Syp mRNA 3′UTR, and the mutation sequence is shown in red. **(B)** The regulatory effect of miR-24-3p on the expression of miR-24-3p was verified by dual-luciferase reporter gene detection. **(C,D)** Representative images and quantification of Syp after the overexpression of miR-24-3p in HT22 cells were tested by western blot. **(E)** The expression level of Syp mRNA was tested by qRT–PCR after the overexpression of miR-24-3p in HT22 cells. The NC mimics group was normalized to 1. **(F,G)** Representative images and quantification of Syp were tested by western blot after cotransfecting Vec-circ-Vps41 and miR-24-3p mimics into HT22 cells treated with D-galactose. **(H)** The expression level of Syp mRNA was tested by qRT-PCR after cotransfecting Vec-circ-Vps41 and miR-24-3p mimics into HT22 cells treated with D-galactose. ^*^p < 0.05, ^**^p < 0.01, ^***^p < 0.001 vs. control + Vector + mimics NC group; ^#^*p* < 0.05, ^##^*p* < 0.01, ^###^*p* < 0.001 vs. D-gal+circ-Vps41 + mimics NC group; The control + Vector + mimics NC group was normalized to 1. Data are shown as the mean ± SEM. *p* values, two-tailed *t*-test, one-way ANOVA. ^*^p < 0.05, ^**^p < 0.01, ^***^p < 0.001.

To unravel the downstream target gene of miR-24-3p, we used a set of online prediction tools: TargetScan, miRBase and RNAhybrid. We detected that the Syp 3′ UTR region contains the conserved MRE of miR-24-3p (Figure 5A). To evaluate whether Syp is the direct target of miR-24-3p, luciferase reporter plasmids with the wild-type Syp mRNA 3′ UTR sequence (Syp 3′ UTR WT) and mutant Syp mRNA 3′ UTR sequence (Syp 3′ UTR Mut) were generated. The results revealed that the transfection of miR-24-3p mimics significantly reduced the luciferase activity of Syp 3′ UTR WT compared with that of Syp 3′ UTR Mut in 293A cells (Figure 5B). Western blot and qRT-PCR analysis showed that the transfection of miR-24-3p mimics significantly reduced Syp protein and mRNA levels *in vitro* (Figures 5C–E).

To further confirm that the improving of Syp levels by circ-Vps41 depends on miR-24-3p, experiments were conducted by co-transfecting Vec-circ-Vps41 and miR-24-3p mimics into HT22 cells treated with D-galactose. The experiments showed that Vec-circ-Vps41 transfection reversed the decreasing of Syp protein and Syp mRNA by the D-galactose treatment in HT22 cell. The miR-24-3p mimics partially abolished the effect of Vec-circ-Vps41 on the expression of Syp in D-galactose-induced aging HT22 cells (Figures 5F–H).

### Overexpression of circ-Vps41 reversed D-galactose-induced learning and memory impairment in mice

**Figure 6:**
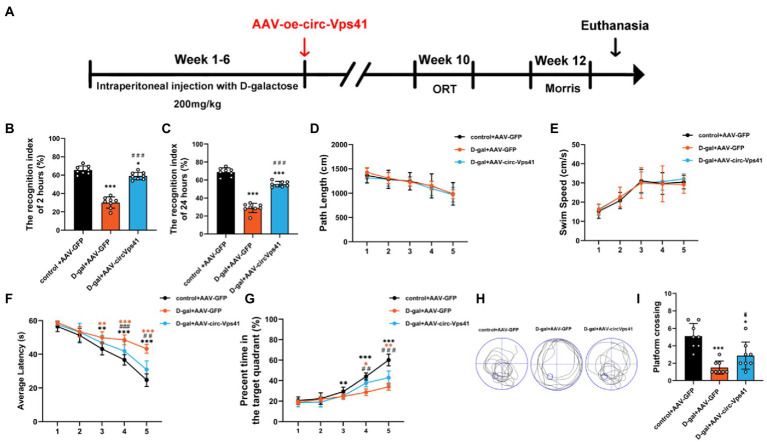
Overexpression of circ-Vps41 reversed D-galactose-induced learning and memory impairment in mice **(A)** Experimental schematic diagram. After 6 weeks of continuous intraperitoneal injection with D-galactose, the adeno-associated virus vector with fluorescence-specific overexpression of circ-Vps41 (AAV-circ-Vps41) and the control GFP were injected by brain stereotactic technology into the hippocampal CA1 area of D-galactose-treated mice. **(B,C)** ORT test of mice at 2 h and 24 h. **(D,E)** Total path length and average swim speed by mice to reach the platform during the training trials. **(F,G)** Time taken by mice to reach the platform and percentage of time in the target quadrants during the training trials. **(H,I)** Representative swimming paths of mice and numbers of platform crossings on Day 7 of the MWM (*n* = 8 for the control+AAV-GFP group, *n* = 8 for the D-gal+AAV-GFP group, *n* = 8 for the D-gal+AAV-circ-Vps41 group). ^*^p < 0.05, ^**^p < 0.01, ^***^p < 0.001 vs. control+AAV-circRNA NC group; ^#^*p* < 0.05, ^##^*p* < 0.01, ^###^*p* < 0.001 vs. D-gal+AAV-GFP group; Data are shown as the mean ± SEM. *p* values, two-tailed *t*-test, one-way ANOVA, repeated measures and multivariate analysis of variance.

To more comprehensively confirm the protective effect of circ-Vps41 in the D-galactose-induced aging model, we investigated the effects of circ-Vps41 overexpression on the hippocampus-dependent learning and memory *in vivo* (Figure 6A). ORT assays revealed that overexpression of circ-Vps41 in D-galactose-induced aging mice increased the recognition index after 2 h (Figure 6B) and 24 h (Figure 6C). In MWM test, all groups showed no significant differences in the total path (Figure 6D) and average swimming speed (Figure 6E), indicating that there were no visual and motion disorders in the mice. The escape latency gradually shortened in all groups. The escape latency in the D-gal+AAV-GFP group was obviously longer compared with the control group and significantly shorter in the D-gal+AAV-circ-Vps41 group when compared to the D-gal+AAV-GFP group from day 3 (Figure 6F). The percentage of time in the target quadrant in the D-gal+AAV-circ-Vps41 group significantly longer when compared to the D-gal+AAV-GFP group from day 4 (Figure 6G). After the acquisition training, we removed the platform to assess the spatial memory. Our results revealed that the D-gal+AAV-circ-Vps41 group and control group spent had more crossing numbers compared with D-gal+AAV-GFP group (Figures 6H-I). In summary, these data indicated that overexpression of circ-Vps41 in D-galactose-induced aging mice significantly prevented aging related impairment in learning and memory.

## Discussion

**Figure 7:**
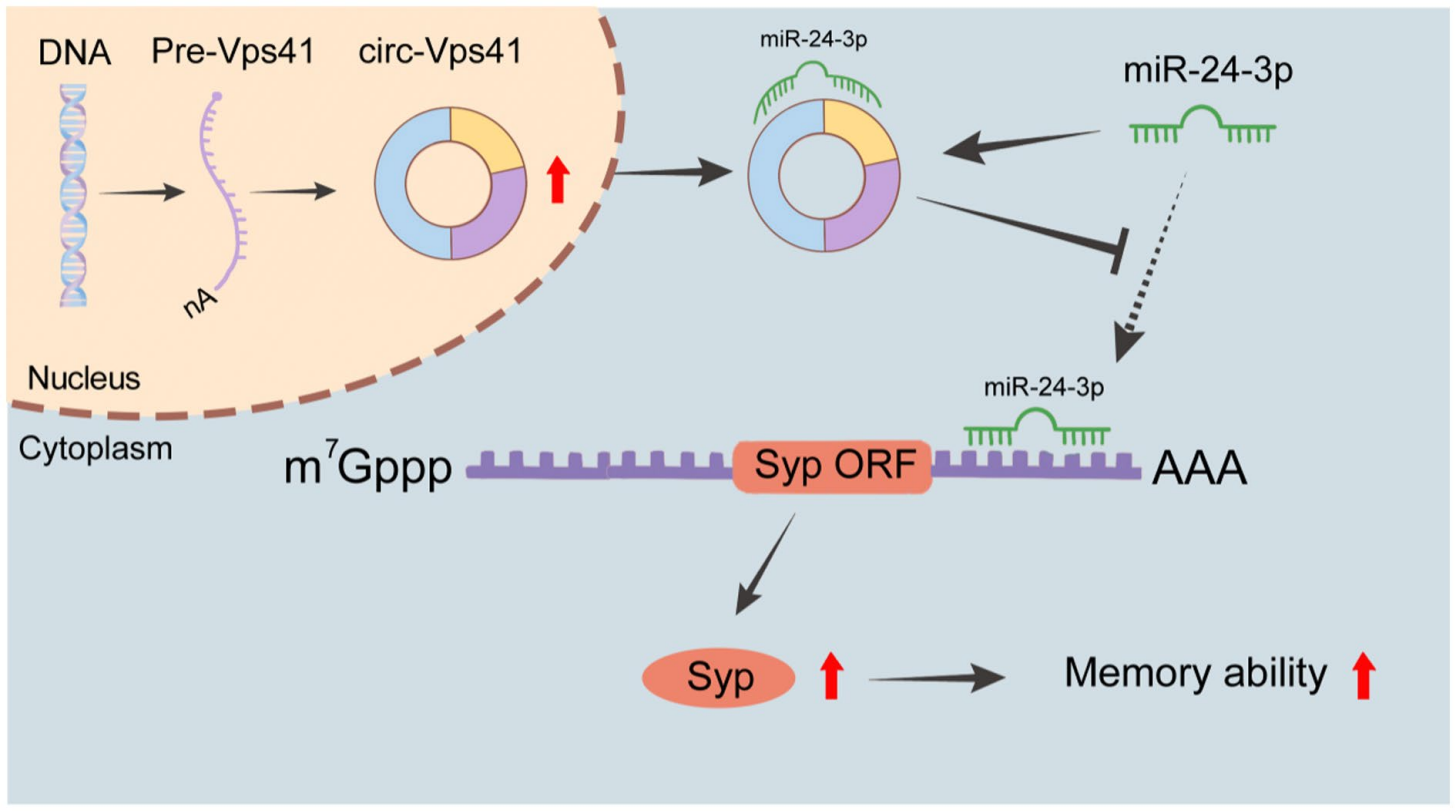
Schematic representation of the molecular mechanisms. circ-Vps41 acts as a competitive endogenous RNA and regulates the expression of Syp by sponging miR-24-3p, ultimately improving the learning and memory of aging mice.

In this study, we found that circ-Vps41 expression was significantly reduced in D-galactose-induced aging model *in vitro* and *in vivo*. Overexpression of circ-Vps41 could improve the expression of Syp, increase the dendritic spine density and improves aging-related learning and memory disorders in D-galactose-induced aging mice. Further studies showed that circ-Vps41 improves aging-related learning and memory disorders *via* the miR-24-3p/Syp axis (Figure 7).

CircRNA has important biological functions, which can regulate gene expression at transcription, post-transcription and translation levels (Zhou et al., 2020; Shao et al., 2021). Among them, circRNAs have many miRNA binding sites to bind competitively to miRNAs, thus inhibiting the binding of miRNA to target gene mRNA (Ren et al., 2020). It is one of the classic mechanisms for circRNAs to exert their functional effects, especially for those that are located within cytoplasm (Chen et al., 2019). In previous studies, we found that circ-Vps41 was mainly located in the cytoplasm (Zhang et al., 2022), indicating that circ-Vps41 may regulate gene expression at the post-transcriptional level. Based on the bioinformatics analysis, we found that circ-Vps41 harbors the miR-24-3p target sites. Combining with dual-luciferase reporter experiments shows that circ-Vps41 binds competitively to miR-24-3p as a sponge. Furthermore, we observed that the miR-24-3p was negatively regulated by circ-Vps41 in the D-galactose-induced aging model, suggesting that miR-24-3p may participate in the process of aging mediated by circ-Vps41. Additionally, luciferase reporter assay displayed direct interaction between circ-Vps41 and miR-24-3p. These findings suggest that circ-Vps41 may exert its function in the process of aging by adsorbing to miR-24-3p.

Previous studies have confirmed that microRNAs regulate gene expression by binding to complementary target mRNAs and promoting their decay or inhibiting their translation (Mansini et al., 2018). Syp was predicted by several online databases as the target of miR-24-3p. It has been reported that downregulation of miR-24-3p by an antisense inhibitor promoted neurite outgrowth as well as levels of Syp expression (Kang et al., 2019). In this study, overexpression of miR-24-3p inhibited expression of Syp. Luciferase reporter gene experiments show that miR-24-3p directly targets the 3′ UTR of Syp. Moreover, the results of rescue tests mentioned that the protective action of circ-Vps41 overexpression was weaken with the miR-24-3p mimics *in vitro*. This indicates that circ-Vps41 regulates Syp expression by sponging miR-24-3p.

The expression of Syp in hippocampus is closely related to learning and memory (VanGuilder et al., 2011; Ma et al., 2019). Syp levels show age-dependent changes in the hippocampus and cortex of mice, and these changes are associated with cognitive performance (Eastwood et al., 2006; Haley et al., 2010). Studies have shown that Syp can promote the release of intersynaptic transmitters (Mullany and Lynch, 1998) and enhance the efficiency of intersynaptic transmission (Lu and Chow, 1999). The Syp/Syngr1 double KO mice showed impaired long-term potentiation (Janz et al., 1999). In contrast, Activation of Syp expression in the hippocampus of SAMP8 mice can improve their ability of learning and memory, memory retention and relearning (Zhou et al., 2018). It has been confirmed that increased expression of Syp was related to improved long-term memory due to enhanced synaptic plasticity (Wi et al., 2016). In this study, we found that the expression of Syp in the hippocampus of D-galactose-induced aging mice was significantly decreased. Overexpression of circ-Vps41 could significantly increase the expression of Syp, promote the density of dendritic spines in the hippocampus, and ultimately promoting learning and memory in D-galactose-induced aging mice. This result is consistent with an earlier study that circ-Vps41 improve learning and memory impairment in Nrf2 knockout mice by our team (Zhang et al., 2022).

In conclusion, the current study provide consistent evidence demonstrating that the overexpression of circ-Vps41 potentially improves the learning and memory in aging *via* Syp upregulation through the miR-24-3p. This study provides a new reference for understanding the molecular pathophysiology mechanisms of aging-related memory and offers a potential therapeutic option for memory impairment in aging. However, there are still limitations in the current study. More research is needed to confirm how age affects circ-Vps41 expression, and whether it affects aging-related memory through other targets.

## Data availability statement

Publicly available datasets were analyzed in this study. This data can be found at: https://www.ncbi.nlm.nih.gov/geo/query/acc.cgi?acc=GSE122421; https://www.ncbi.nlm.nih.gov/geo/query/acc.cgi?acc=GSE122422.

## Ethics statement

The animal study was reviewed and approved by the Ethics Committee of Hebei Medical University (IACUC-Hebmu-Glp-2,016,017).

## Author contributions

DG and LW contributed to the conception and design of the study. YL, RZ, and YG contributed the materials and performed the experiment. QL, ZL, and WX performed the data analyses. YL and HW contributed significantly to the drafting of the manuscript. DG contributed to critical revision of the manuscript. All authors contributed to the article and approved the submitted version.

## Funding

This work was supported by the National Natural Science Foundation of China (Grant No. 82071594), Hebei Medical University research development fund, Natural Science Foundation of Hebei Province (Grant Nos. C2020206037 and H2020206299), and Key Scientific and Technological Research Plan of Hebei Province Medical Science Research Project (Grant Nos. 20212689 and 20212491).

## Conflict of interest

The authors declare that the research was conducted in the absence of any commercial or financial relationships that could be construed as a potential conflict of interest.

## Publisher’s note

All claims expressed in this article are solely those of the authors and do not necessarily represent those of their affiliated organizations, or those of the publisher, the editors and the reviewers. Any product that may be evaluated in this article, or claim that may be made by its manufacturer, is not guaranteed or endorsed by the publisher.

## Footnotes

^1^ http://imagej.net/Fiji
