# Supplementary material for: Circ-Vps41 positively modulates Syp and its overexpression improves memory ability in aging mice
Source: Front Mol Neurosci. 2022 Dec 2;15:1037912. doi: 10.3389/fnmol.2022.1037912 (PMC9756809; doi:10.3389/fnmol.2022.1037912)
Supplement: Supplementary file 1 [file Data_Sheet_1.pdf]

Supplementary Material

1 Supplementary Figures

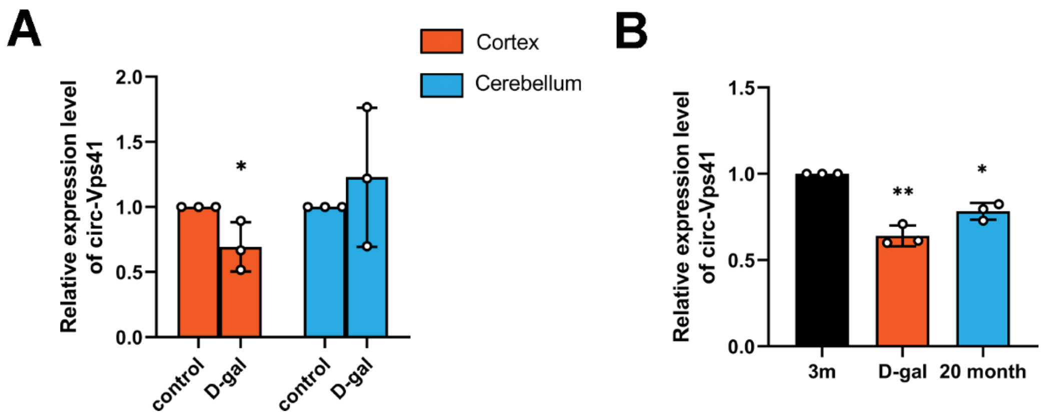

Supplementary Figure 1.

(A)The expression of circVps41 in different regions of brain structure (cortex, cerebellum) in D-galactose-induced aging model mice using qPCR. *$p < 0.05$ vs. control group.

(B) The expression of circ-Vps41 in the hippocampus of 3-month-old, 20-month-old wild-type and D-galactose aging model mice using qPCR. *$p < 0.05$, **$p < 0.01$ vs. 3 m group.

The Control group and 3 m group were normalized to 1. Data are shown as the mean ± SEM. *p* values, two-tailed t test, one-way ANOVA.

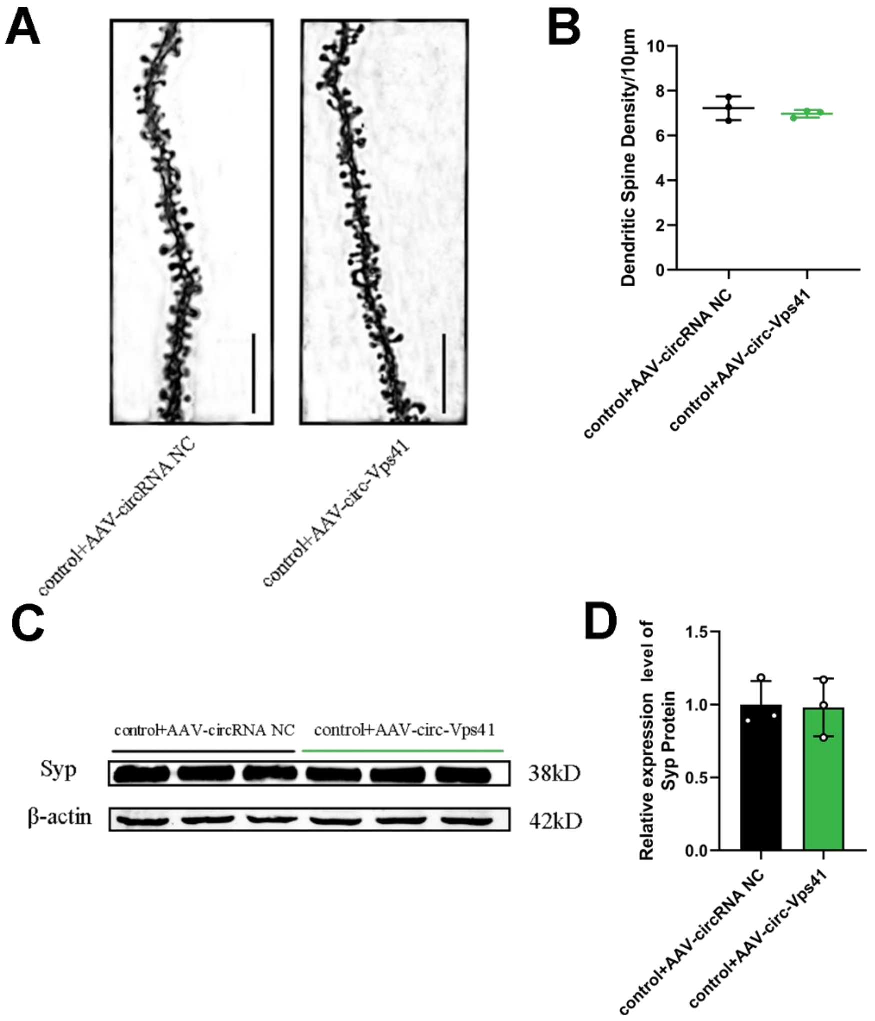

Supplementary Figure 2.

(A, B) Representative images and quantification of Golgi-Cox staining in the hippocampus of the control+AAV-circRNA group and the control + AAV-circ-Vps41 group.

(C, D) Representative images and quantification of the protein expression of Syp in the hippocampus of the control+AAV-circRNA group and the control+AAV-circ-Vps41 group were tested by western blot.

Data are shown as the mean ± SEM. *p* values, two-tailed t test.

2 The full length original western blots

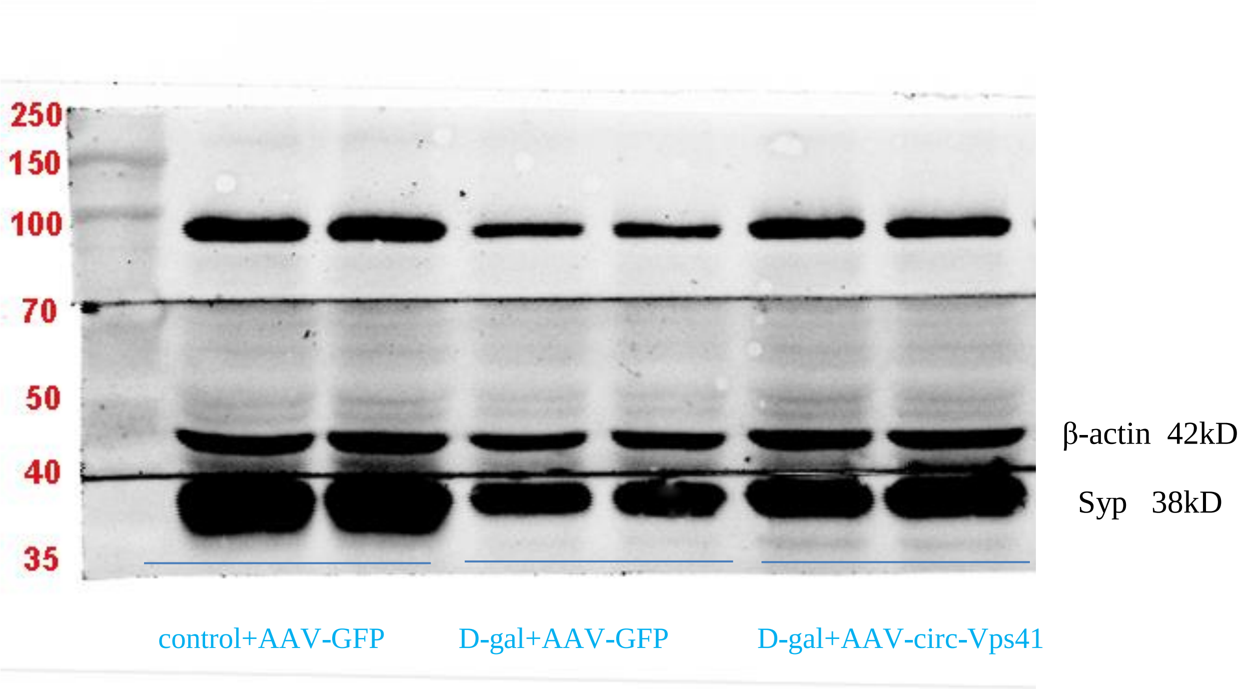

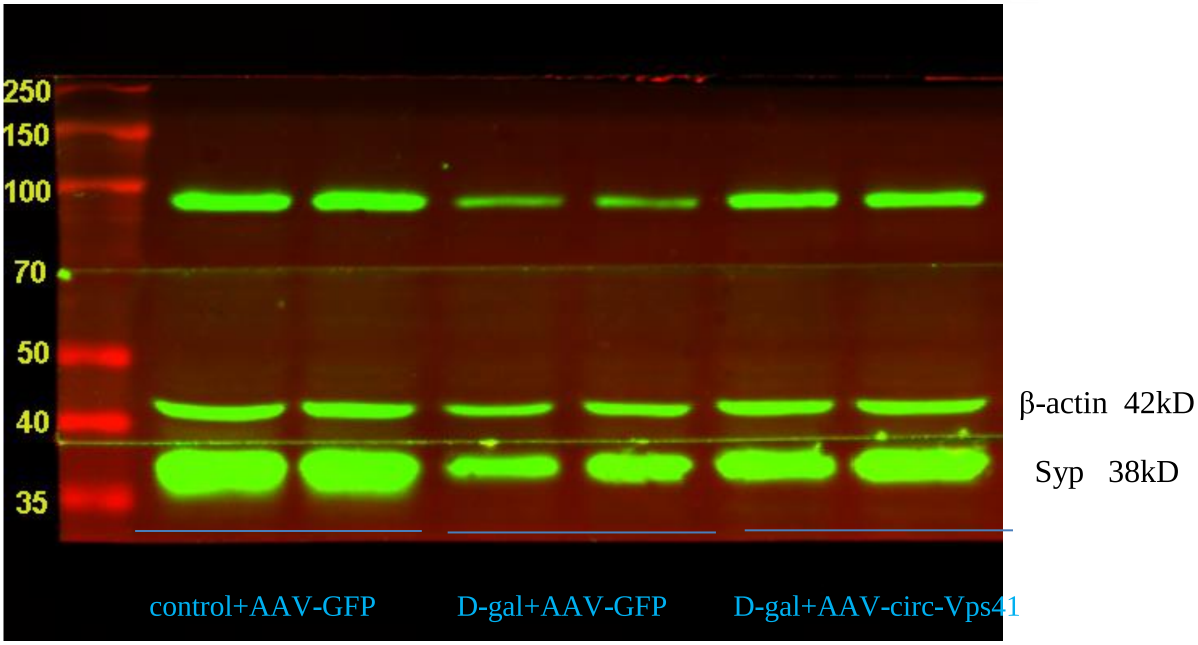

2.1 Figure. 2 E Representative images of Syp in the hippocampus of the 3 groups of mice were tested by western blot.

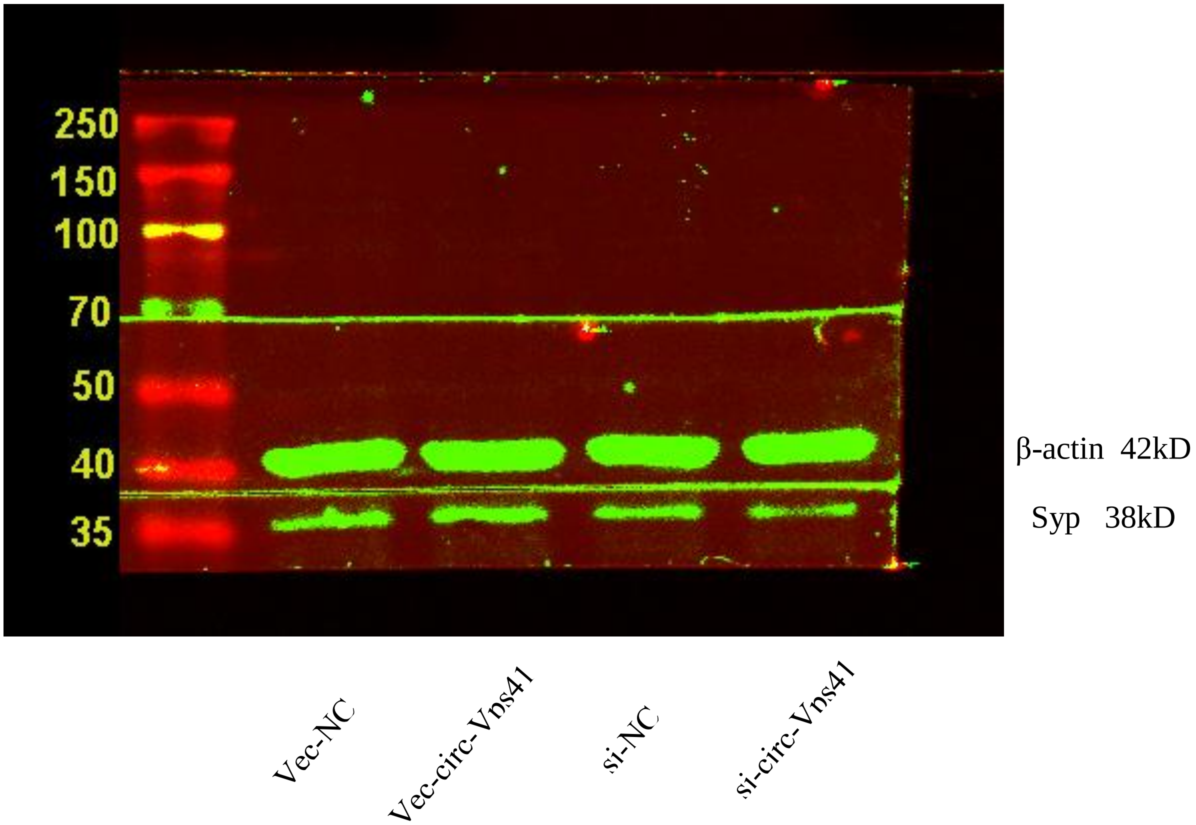

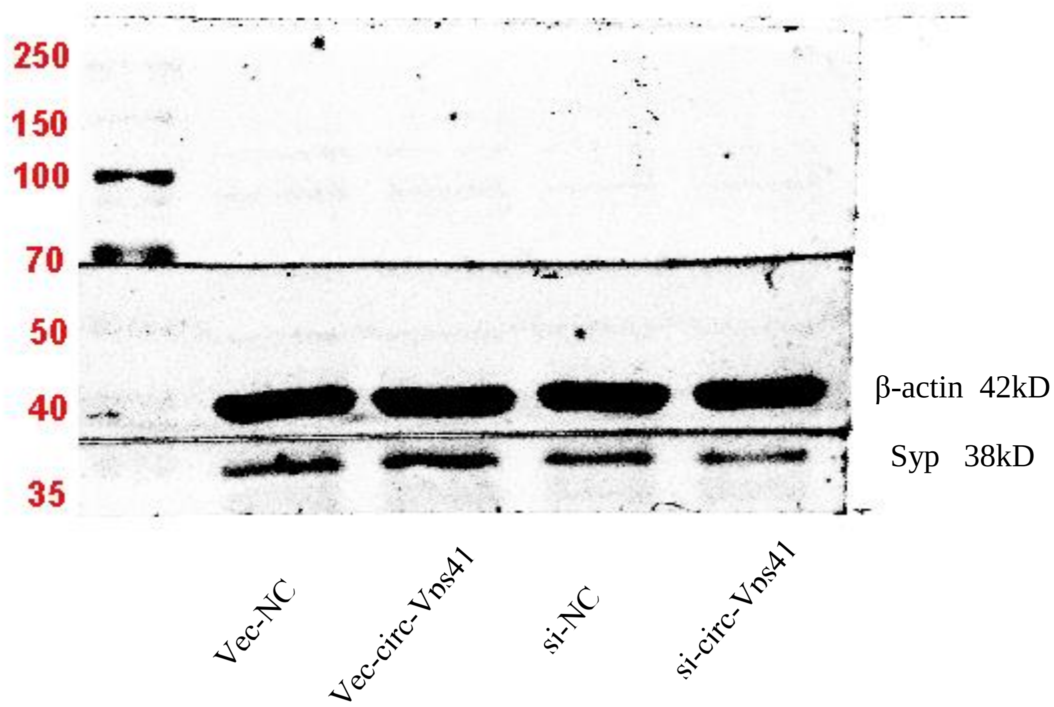

2.2 Figure. 3 B Representative images of Syp in HT22 cells after knockdown and overexpression of circ-Vps41 were tested by western blot.

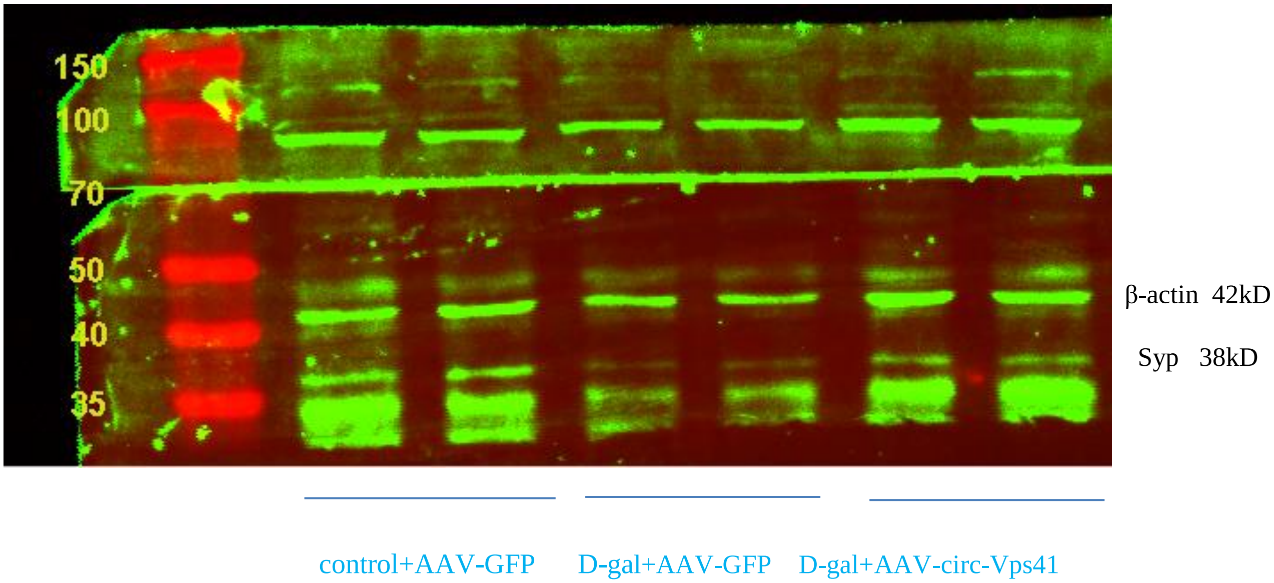

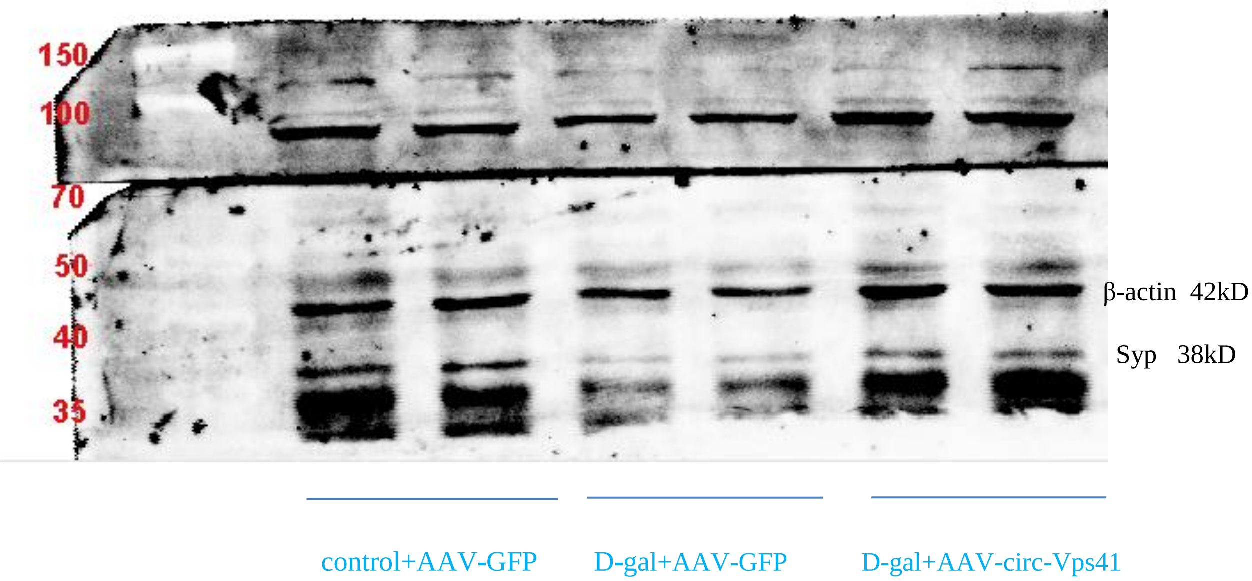

2.3 Figure. 3 F Representative images of Syp after the overexpression of circ-Vps41 in D-galactose-induced aging HT22 cells were tested by western blot.

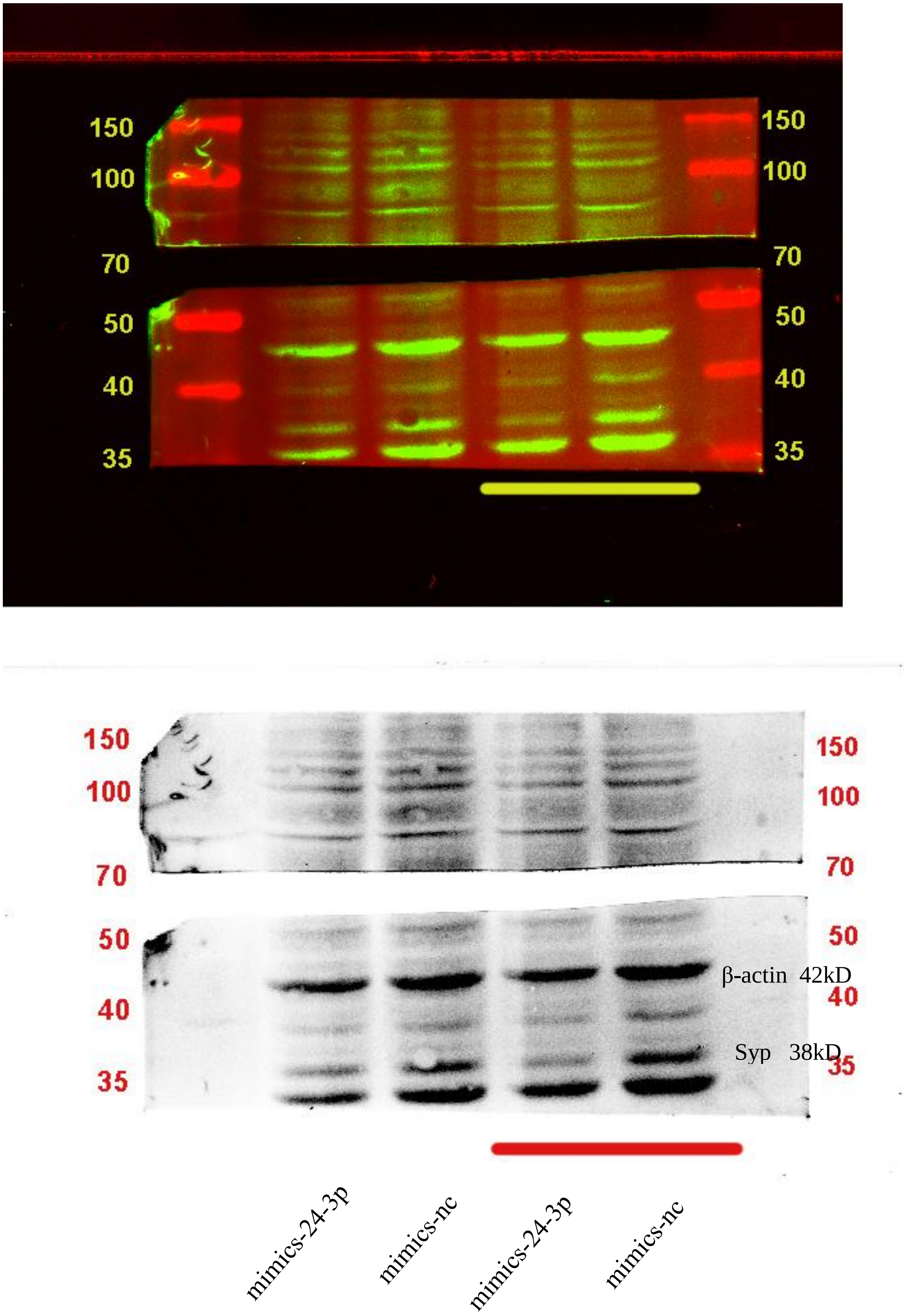

2.4 Figure. 5 C Representative images of Syp after the overexpression of miR-24-3p in HT22 cells were tested by western blot.

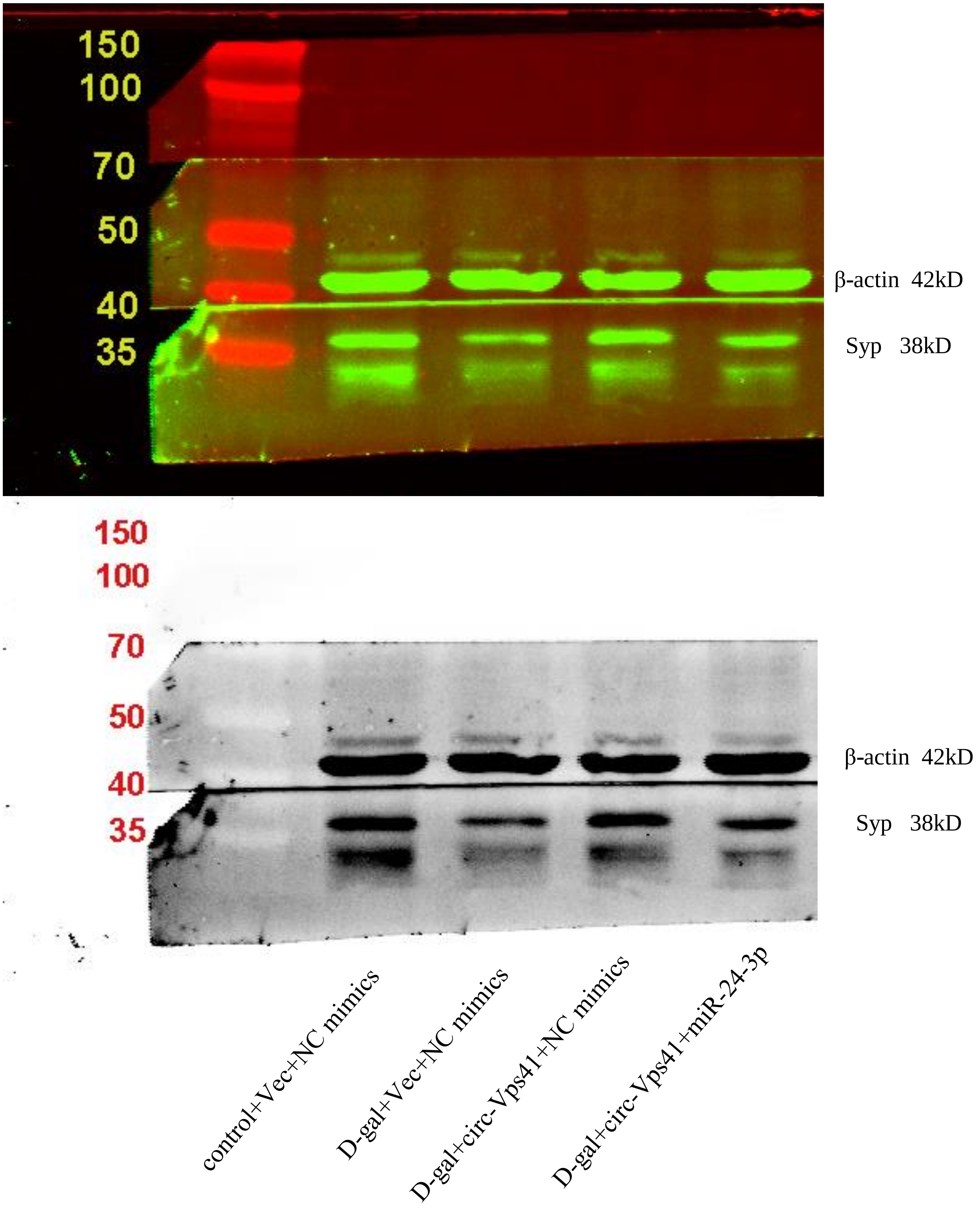

2.5 Figure. 5 F Representative images of Syp were tested by western blot after cotransfecting Vec-circ-Vps41 and miR-24-3p mimics into HT22 cells treated with D-galactose.

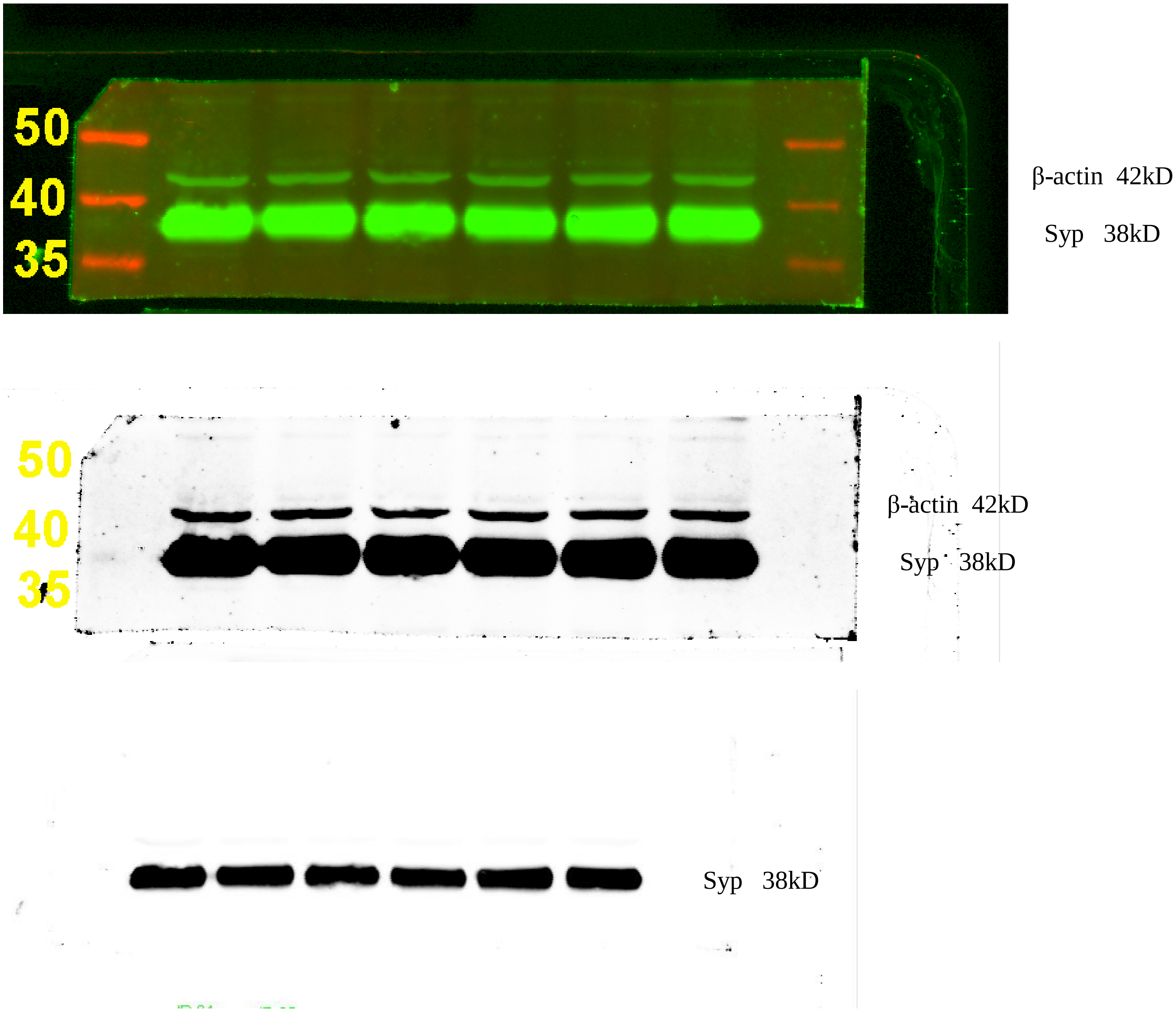

2.6 Supplementary Figure 2. Representative images and quantification of the protein expression of Syp in the hippocampus of the control+AAV-circRNA group and the control+AAV-circ-Vps41 group were tested by western blot.
